# No detectable differences in Nef-mediated downregulation of HLA-I and CD4 molecules among HIV-1 group M lineages circulating in Cameroon, where the pandemic originated

**DOI:** 10.3389/fviro.2024.1379217

**Published:** 2024-05-29

**Authors:** Nelson Sonela, Jaclyn Mann, Celestin Godwe, Oumarou H. Goni, Mérime Tchakoute, Nathalie Nkoue, Tulio de Oliveira, Mark A. Brockman, Zabrina L. Brumme, Thumbi Ndung’u, Marcel Tongo

**Affiliations:** 1 Center of Research for Emerging and Re-Emerging Diseases (CREMER), Institute of Medical Research and Study of Medicinal Plants (IMPM), Yaoundé, Cameroon; 2 Chantal BIYA International Reference Centre for Research on HIV/AIDS prevention and management (CIRCB), Yaoundé, Cameroon; 3 Department of Medicine, Weill Cornell Medical College, Cornell University, New York, NY, United States; 4 HIV Pathogenesis Programme, University of KwaZulu-Natal, Durban, South Africa; 5 Départment of Biochemistry, University of Douala, Douala, Cameroon; 6 Départment of Microbiology, Faculty of Sciences, University of Yaoundé 1, Yaoundé, Cameroon; 7 Programmes de Santé et développement au sein du Groupement de la Filière Bois du Cameroun, Yaoundé, Cameroon; 8 Centre for Epidemic Response and Innovation (CERI), School of Data Science and Computational Thinking, Stellenbosch University, Stellenbosch, South Africa; 9 KwaZulu-Natal Research Innovation and Sequencing Platform (KRISP), School of Laboratory Medicine and Medical Sciences, College of Health Sciences, Nelson R Mandela School of Medicine, University of KwaZulu-Natal, Durban, South Africa; 10 Molecular Biology and Biochemistry, Simon Fraser University, Burnaby, BC, Canada; 11 Faculty of Health Sciences, Simon Fraser University, Burnaby, BC, Canada; 12 British Columbia Centre for Excellence in HIV/AIDS, Vancouver, BC, Canada; 13 Max Planck Institute for Infection Biology, Berlin, Germany; 14 Africa Health Research Institute (AHRI), Durban, South Africa; 15 Ragon Institute of Massachusetts General Hospital, Massachusetts Institute of Technology and Harvard University, Cambridge, MA, United States; 16 Division of Infection and Immunity, University College London, London, United Kingdom

**Keywords:** HIV-1 group M, HIV-1 Nef, HLA-I downregulation, CD4 downregulation, Cameroon

## Abstract

HIV-1 group M (HIV-1M) lineages downregulate HLA-I and CD4 expression via their Nef proteins. We hypothesized that these Nef functions may be partially responsible for the differences in prevalence of viruses from different lineages that co-circulate within an epidemic. Here, we characterized these two Nef activities in HIV-1M isolates from Cameroon, where multiple variants have been circulating since the pandemic’s origin. Single HIV-1 Nef clones from 234 HIV-1-ART naïve individuals living in remote villages and two cosmopolitan cities of Cameroon, sampled between 2000 and 2013, were isolated from plasma HIV RNA and analyzed for their capacity to downregulate HLA-I and CD4 molecules. We found that, despite a large degree of within- and inter- lineage variation, the ability of Nef to downregulate HLA-I was similar across these different viruses. Moreover, Nef-mediated CD4 downregulation activity was also well conserved across the different lineages found in Cameroon. In addition, we observed a trend towards higher HLA-I downregulation activity of viruses circulating in the cosmopolitan cities versus the remote villages, whereas the CD4 downregulation activities were similar across the two settings. Furthermore, we noted a significant decline of HLA-I downregulation activity from 2000 to 2013, providing additional evidence supporting the attenuation of the global HIV-1M population over time. Finally, we identified 18 amino acids associated with differential HLA-I downregulation and 13 amino acids associated with differential CD4 downregulation within the dominant CRF02_AG lineage. Our lack of observation of HIV lineage-related differences in Nef-mediated HLA-I and CD4 downregulation function suggests that these activities do not substantively influence the prevalence of different HIV-1M lineages in Cameroon.

## Introduction

HIV-1 Nef, a 27-35 kDa protein highly expressed during the early stages of the HIV-1 life cycle, promotes HIV-1 pathogenesis and immune evasion (1–11). Two of Nef’s main functions are to downregulate HLA-I and CD4 molecules from the cell surface (7, 9, 12–16). Downregulation of HLA-I molecules allows HIV-1-infected cells to evade immune recognition by CD8+ T cells (17), while CD4 downregulation enhances HIV-1 infection, replication (1, 15, 16, 18–20). Specifically, while HIV’s entry receptor CD4 is indispensable for viral infection of CD4+ T-cells, its continued presence on the cell surface disrupts the processing of viral glycoproteins and reduces Envelope (Env) incorporation into virions, impeding their release and infectivity (19–22). CD4 downregulation by Nef is also thought to prevent superinfection (10, 14, 23) and allows the virus to avoid antibody-dependent cell cytotoxicity by abrogating CD4-induced Env conformational changes required for antibody binding (24)

The HIV-1 *nef* gene is highly polymorphic, with nucleic acid sequence diversity ranging from 14.4% to 23.8% between different HIV group M subtypes (HIV-1M) (25). There is also evidence that Nef function may differ between some of the major HIV-1M subtypes (6, 26). For example, Yoon et al. (27) showed that HIV-1 Nef clones derived from HIV-1M subtype B isolates downregulated HLA-I significantly better than those derived from subtype D isolates, whereas Nef clones derived from subtype D isolates downregulated CD4 significantly more than those derived from subtype B isolates (27). Also, Turk et al. (28) observed that Nef closes from subtype B and F isolates downregulated HLA-I significantly more than those from subtype C isolates (28). Finally, Mann et al. (15) showed that the HLA-I and CD4 downregulation abilities of Nef differ among HIV-1M subtypes A, B, C, and D (15) and that these functions correlated with markers of disease progression (16). These findings suggest that variation in HIV-1 Nef functions among subtypes may contribute to global differences in viral pathogenesis and spread. However, most of these studies were restricted to *nef* genes sampled from viruses belonging to the major HIV-1M subtypes A, B, C, and D, and thus did not fully reflect HIV-1M global diversity (15, 16, 27, 28).

Cameroon was the likely site of cross-species transmission that yielded HIV-1 group M (HIV-1M) (29, 30). Possibly because of this, Cameroon, like other countries in the Cong Basin, has one of the genetically most diverse HIV epidemics in the world (31–33). Despite the extensive characterization of HIV-1M diversity in Cameroon and the identification of multiple highly divergent HIV-1M lineages, primarily complex unique recombinant forms and rare variants (33, 34), the phenotypic properties of these lineages are almost entirely unknown. Such rare viruses may have unique biological characteristics, some of which may help understand why these lineages have remained restricted to Cameroon. It is, therefore, tempting to speculate that among the large pool of HIV-1M lineages that are presently circulating within Cameroon, most of them fail to reach some required threshold for HLA-I and CD4 downregulation capacity that would enable them to spread and cause large sub-epidemics within Cameroon and elsewhere. Alternatively, the diversity of HIV-1M lineages in Cameroon may also be consistent with the hypothesis that, possibly even before the onset of the global pandemic, multiple HIV-1M lineages co-circulated within Cameroon; and that the present-day frequency differences between lineages in Cameroon reflect a composite of slight differences in the times when lineages first arrived (or emerged) in Cameroon. Here, we measured the ability of donor-derived Nef isolates to downregulate HLA-I and CD4 to test the hypothesis that varying Nef-driven pathogenicity is associated with the circulating frequencies of HIV-1M lineages in Cameroon. We additionally wished to evaluate the impact of specific Nef polymorphisms on the CD4 and HLA-I downregulation abilities of this protein.

## Materials and methods

### Study participants

Plasma samples were collected anonymously and voluntarily from ART-naïve individuals living with HIV and residing in two cosmopolitan cities and 40 remote villages located in the equatorial rain forest in Cameroon from 2000 to 2013. Samples in remote villages were collected in 2000, 2012 and 2013, while those in the cities were sampled in 2007, 2008 and 2009. This study was approved by the Cameroonian National Ethics Committee (ethical clearance number: 2019/04/1156/CE/CNERSH/SP). Viral loads were measured at one time point before treatment initiation. The characterization of the genetic diversity of HIV-1M strains circulating in these cohorts has been described in (33, 35).

### Preparation of HIV-1 Nef clones

HIV RNA was extracted from stored plasma using the QIAamp^®^ Viral RNA Extraction Kit (Qiagen, Hilden, Germany) according to manufacturer protocols. Complementary DNA (cDNA) was generated using the ImProm-II™ Reverse Transcription System kit (Promega, Madison, Wisconsin, USA), also according to the manufacturer’s protocol. Following this, the entire *nef* region was amplified by nested PCR (ROCHE Expand High Fidelity kit; Roche-Mannheim, Germany) using non-subtype-specific HIV-1 group M primers. The PCR cycling conditions were the same for both the first and second rounds, consisting of 94°C for 2 min followed by 35 cycles of 94°C for 15 s, 55°C for 15 s, 72°C for 50 s and then 72°C for 7 min. The first round primers were: Nef outer5-le (HXB2: 8513 – 8533; 5’-GTGCCTCTTCAGCTA CCACCG-3’ and Nef outer3-3e (HXB2: 9488 – 9508; Reverse primer 5’-AGCATCTGAGGGTT AGCCACT-3 ’). The *nef* second round primers were: NEF8746_Sgrl_Ascl_F (HXB2: 8736-8772; 5’- AGAGC ACCGGCGCGCCTCCACATACCTASAAGAATMAGACARG-3’) and Nef 9474_Sacll_Clal_R 3-7e (HXB2: 9449-9491; 5’-GCCT CCGCGGATCGATCAGGCCACRCCTCCCTGGAAASKCCC-3’) (35). The second-round primers contain restriction sites AscI (forward) and SacII (reverse), shown in bold, to facilitate downstream cloning experiments. Each donor-derived HIV-1 *nef* amplicon was purified using the QIAQuick PCR purification kit (Qiagen, Germany), digested with AscI and SacII restriction enzymes at 37 °C for 2 hours and cloned into a pSELECT-GFPzeo expression plasmid (Invitrogen, Canada) that was modified to contain AscI and SacII restriction sites. The pSELECT-GFPzeo plasmid contains two transcription units. The first expression cassete with hEF1/HTLV promoter drives the expression of the inserted nef gene while, the second unit with the CMV/HTLV promoter drives the expression of the GFP-zeo gene (36, 37). The digested plasmid and nef amplicon were ligated and ligation mixture was transformed into OneShot TOP10 competent cells (Invitrogen, Canada) according to the manufacturer’s instructions. The transformed cells were plated onto Luria-Bertani agar plates containing zeocin and incubated at 37 °C overnight for 16 hours. Colonies were picked, boiled at 95 °C for 10 minutes in 10μl of nuclease-free water, and used as templates in colony PCR to confirm the presence of the *nef* gene. The bulk plasma-derived *nef* PCR products and the *nef* clones amplified by colony PCR were sequenced using the ABI Prism Big Dye Terminator v3.1 Sequencing Kit (Applied Biosystems, USA). The resulting bulk and clonal *nef* sequences were aligned using MEGA version 10 (38) and a maximum likelihood tree was constructed using IQ Tree using the best fit GTR+I+G nucleotide substitution model (http://iqtree.cibiv.univie.ac.at). Branch support values were computed through IQ Tree’s ultrafast bootstrap analysis (1000 resampling iterations) with Shimodaira-Hasegawa approximate likelihood ratios ([SH]-aLRT) ≥ 0.99] (39). The phylogenetic trees were rooted using an HIV-1 group P sequence (35) and visualized using Figtree v1.4.4 (40). The *nef* clone sequences were submitted to GenBank under the accession numbers OR979846 - OR980068.

### HLA-I and CD4 downregulation assays

Nef-mediated HLA-I and CD4 downregulation ability was measured as previously described (15). Briefly, an immortalized CD4+ T cell line previously modified to express high levels of HLA-A*02 (CEM-A*02) (15) was used to measure the HLA-I and CD4 downregulation activity of each Nef clone. The cells were maintained in R10 medium (RPMI-1640 supplemented with 10% FBS, 1% L-glutamine, 1% HEPES buffer, and 0.5% penicillin-streptomycin). A total of 6 x 10^5^ CEM-A*02 cells in MegaCell medium (Sigma, USA) were transfected with 8000 ng of Nef clone by electroporation (BioRad Gene Pulser Xcell Electroporation system). The transfected cells were supplemented with R10 and incubated for 20 hours overnight. The cells were stained with APC-labeled anti-CD4 and PE-labeled anti-HLA-A*02 antibodies (BD Biosciences, USA). The surface expression of CD4 and HLA-I were measured by flow cytometry. To determine the relative HLA-I or CD4 downregulation ability of each donor-derived Nef clone, the median fluorescence intensity (MFI) of CD4 or HLA-I expression in GFP-positive (*i.e*. Nef-expressing) cells was normalized to the MFI of HLA-I or CD4 expression in the negative control (empty pSELECT-GFPZeo plasmid) and the positive control (Nef from the HIV subtype B SF2 reference strain cloned into pSELECT-GFPZeo) using the following formula = *[(Negative control* – *Donor Nef)/(Negative control* – *Positive control)] x 100*%. A normalized value of 0% indicates no downregulation activity, while a value of 100% indicates a downregulation capacity equivalent to that of the positive control. We performed all assays in duplicate, and the results were presented as the mean of these two measurements.

### Site-directed mutagenesis

The Nef mutation I43V was introduced into a participant-derived CRF02_AG *nef* sequence (BS21, GenBank accession JX244966) chosen for its high amino acid similarity to the consensus CRF02_AG sequence. Briefly, the BS21 sequence was cloned into a TOPO TA 3.1 plasmid (Invitrogen, San Diego, USA) and modified by site-directed mutagenesis using the QuikChange II XL Site-Directed Mutagenesis kit (Agilent Technologies, Texas, USA). Following sequence confirmation, the mutant *nef* was cloned into pSELECT-GFPzeo to assess CD4 and HLA-I down-regulation analysis as described above. Three independent HLA-I and CD4 down-regulation assays were performed.

### Data analysis

We used Kruskal-Wallis tests to compare Nef-mediated HLA-I and CD4 downregulation abilities across all HIV-1M lineages and across different years of sampling, and Mann-Whitney U tests for two-group comparisons. We used Spearman’s correlation to assess the relationships between Nef-mediated activities and log_10_ plasma viral load and between HLA-I and CD4 downregulation capacities. For the set of CRF02_AG Nef clones, we performed a codon-by-codon sequence-function analysis which is a technique that independently looks at each coordinate and determines whether the presence or absence of a particular amino acid is significantly associated with a change in function, to identify amino acid variants associated with significantly increased or decreased Nef-mediated HLA-I and CD4 downregulation ability. In this analysis, multiple comparisons were addressed using q-values (41). Functional comparisons between parental and mutant Nefs were performed using Unpaired t tests. Overall, p-values <0.05 were considered significant.

## Results

### Study participants

Two hundred and thirty-four (234) participants were enrolled between 2000 and 2013. Of these, 125 individuals were recruited in the cosmopolitan cities of Yaoundé (the country’s capital) and Douala (the country’s economic hub), 109 were enrolled from communities in remote locations within the equatorial rain forest. Biological gender data were available for only 103 of the participants; 55 in the cities (amongst whom 41 male and 14 female) sampled in 2007 and 48 in the remote villages (17 males and 41 female) collected in 2000; 101 participants reported their age and the median was 31 years (inter-quartile range [IQR] 27-38) for the cities cohort and 32 [IQR 26-41] for the second cohort. The median plasma HIV-1 RNA viral load was 4.41 log_10_ copies/ml [IQR 3.7-4.9], available for 187 participants; 4.4 [IQR 3.8-4.9] in the cities and 4.5 [IQR 4.0-5.0] in remote villages (Table 1).

The statistically differences in gender, age and the plasma HIV-1 RNA viral loads of individuals living in cosmopolitan cities versus remote communities are summarized in Table 1. All individuals were ART-naïve, and HIV infection dates were not known.

### Selection and functional assessment of Nef clones

**Figure 1:**
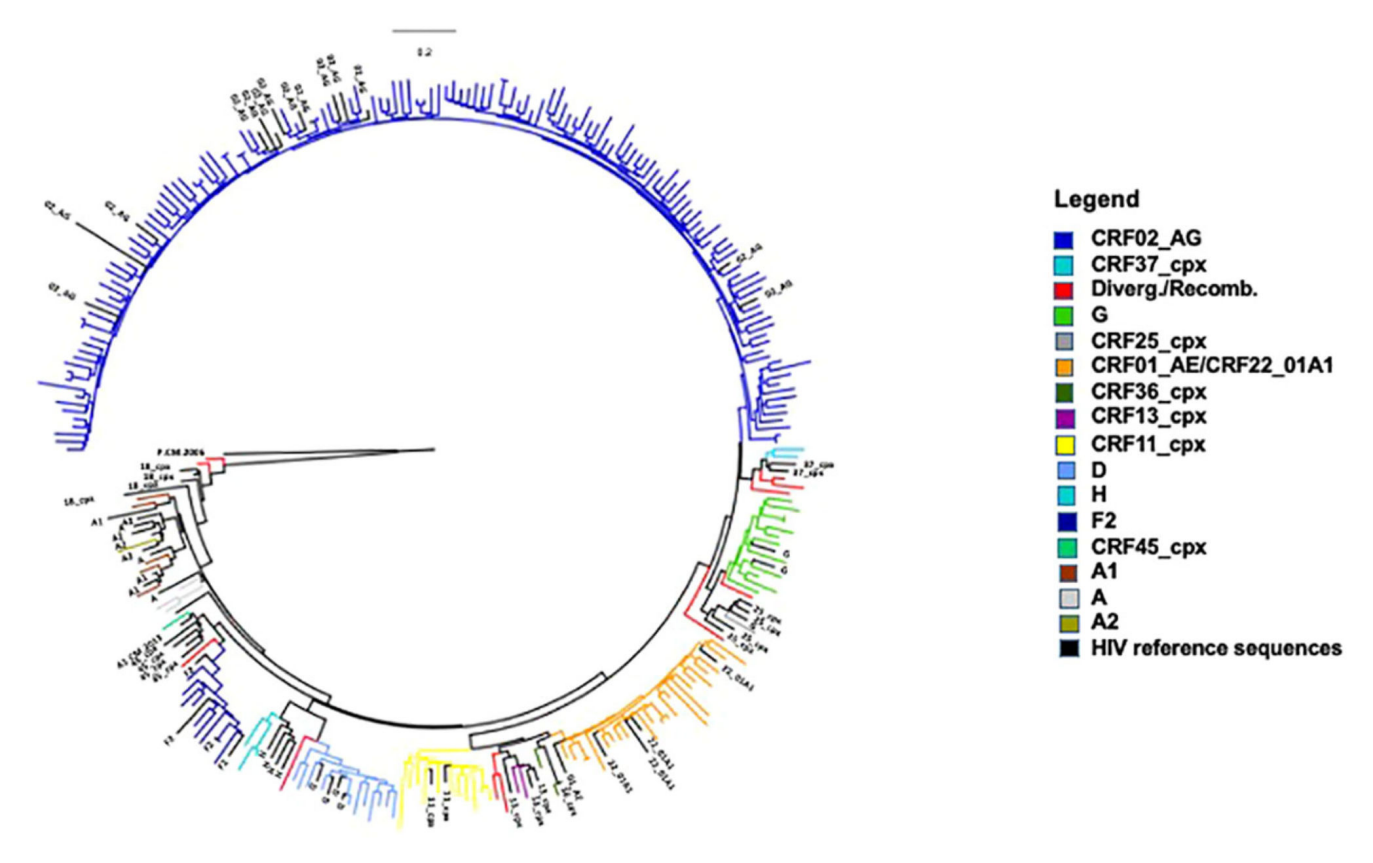
Genetic diversity of HIV-1 group M Nef clones from Cameroon. Maximum-likelihood phylogenetic tree of 234 *nef* clone sequences. The tree was constructed with 1000 full maximum likelihood bootstrap replicates using IQ Tree and using the best fit GTR+I+G nucleotide substitution model (http://iqtree.cibiv.univie.ac.at). Each HIV-1 group M lineage is represented by a unique color. The tree was rooted with an HIV-1 group P sequence.

We have previously characterized HIV-1 group M (HIV-1M) genetic diversity in this cohort (35, 42). Overall, CRF02_AG accounted for 59% of all *nef* clones (n=137), with the remaining 41% (n=97) comprising various subtypes, sub-subtypes and circulating recombinant forms (CRFs) observed at frequencies of 1% to 9%. These included subtypes A (n=3), G and D (n=12 each), and H (n=3); sub-subtypes A1 (n=5), A2 (n=1), and F2 (n=9); and CRFs 01_AE/22_01A1 (n=22), 11_cpx (n=11), 13_cpx (n=2), 25_cpx (n=1), 36_cpx (n=2), 37_cpx (n=2), 45_cpx (n=1). Finally, variants that could not be classified into any known subtypes or CRFs were classified as either divergent or recombinants (n=11) (Figure 1). It should be noted that CRF22_01A1 sequences are embedded within the CRF01_AE cluster in the *nef* gene. In addition, all these isolated Nef clones clustered with their respective bulk plasma sequences (Supplementary Figure 1) and were free of gross genetic defects. Each Nef clone was transfected into the CEM-A*02 T cell line (15), after which, Nef-mediated HLA-A*02 and CD4 downregulation was measured by flow cytometry and normalized to that of the control subtype B strain SF2 (representative data in Supplementary Figures 2A–F). Replicate measurements were consistent for HLA-I and CD4 downregulation (Spearman’s, r=0.85 and p < 0.0001; not shown). Overall, the HLA-I downregulation functions of the 234 isolated Nef clones ranged from 5% to 106% relative to SF2 (median 63%; IQR 45% - 83%) (Supplementary Figure 2G) while their CD4 downregulation function ranged from 9% to 108% relative to SF2 (median 94%; IQR 82% - 99%) (Supplementary Figure 2H). HLA-I downregulation significantly correlated with CD4 downregulation (Spearman’s, r=0.48 and p<0.0001; Supplementary Figure 3).

### Inter-lineage comparison of Nef-mediated HLA-I and CD4 downregulation among viruses circulating in Cameroon.

**Figure 2:**
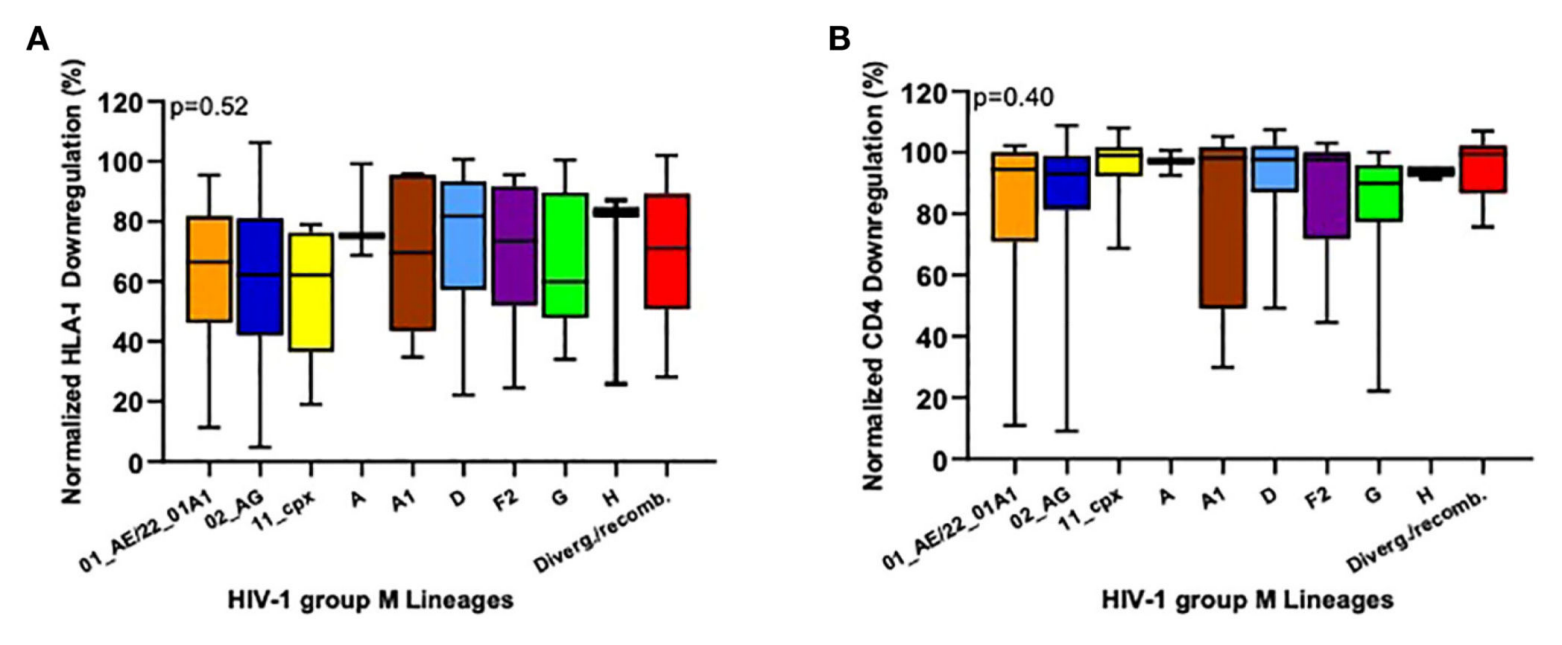
Inter-subtype comparison of HIV-1 Nef-mediated HLA-I and CD4 downregulation abilities. HLA-I **(A)** and CD4 **(B)** downregulation activities were compared between the different HIV-1 group M lineages circulating in Cameroon. Only lineages with at least three representatives are shown. Colors match those in Figure 1. Variants that could not be classified into any known subtypes and recombinant forms were classified as Divergent/Recombinants (Diverg./Recomb.). Horizontal line and error bars represent the median and interquartile range, respectively. We used the Kruskal-Wallis tests to compare Nef functions across lineages.

Previous data have shown that Nef function differ across HIV-1M lineages (43). To detect these differences in our cohort, we stratified our Nef-mediated HLA-I and CD4 downregulation data by HIV-1M lineage (where we only considered lineages with at least three representatives). Though HLA-I downregulation activities varied markedly among the isolates tested, we observed no overall significant differences in this function across the HIV-1M lineages represented in our cohort (p=0.52; Kruskal-Wallis test, Figure 2A). Nevertheless, it was interesting that subtype H lineages exhibited the highest average Nef-mediated HLA-I downregulation ability (median 83%; IQR 26% - 87%) and subtype G the lowest (median 60% and IQR 48% - 90%). Somewhat in contrast, Nef-mediated CD4 downregulation showed a much narrower range of activity between isolates, with the majority displaying normalized function close to 100%. Similar to our observations for HLA-I downregulation, we observed no overall significant differences in CD4 downregulation function across the HIV-1M lineages in our cohort (p=0.40; Kruskal-Wallis’s test, Figure 2B).

Categorizing our samples based on whether or not they belonged to the main circulating CRF02_AG lineage revealed no significant differences in the Nef-mediated receptor downregulation between the two groups (Kruskal-Wallis, p=0.12 for HLA-I and p=0.19 for CD4 downregulation; Supplementary Figures 4A, B). Plasma viral loads (VL) also did not significantly correlate with either Nef-mediated HLA-I or CD4 downregulation activities (Spearman’s correlation, r=0.07 and p=0.36 for HLA-I and r=-0.10 and p=0.17 for CD4 downregulation; Supplementary Figures 5A, B). Furthermore, analyses of the Nef functions according to the sex revealed no significant differences (data not shown).

### Temporal trends in Nef function over time

**Figure 3:**
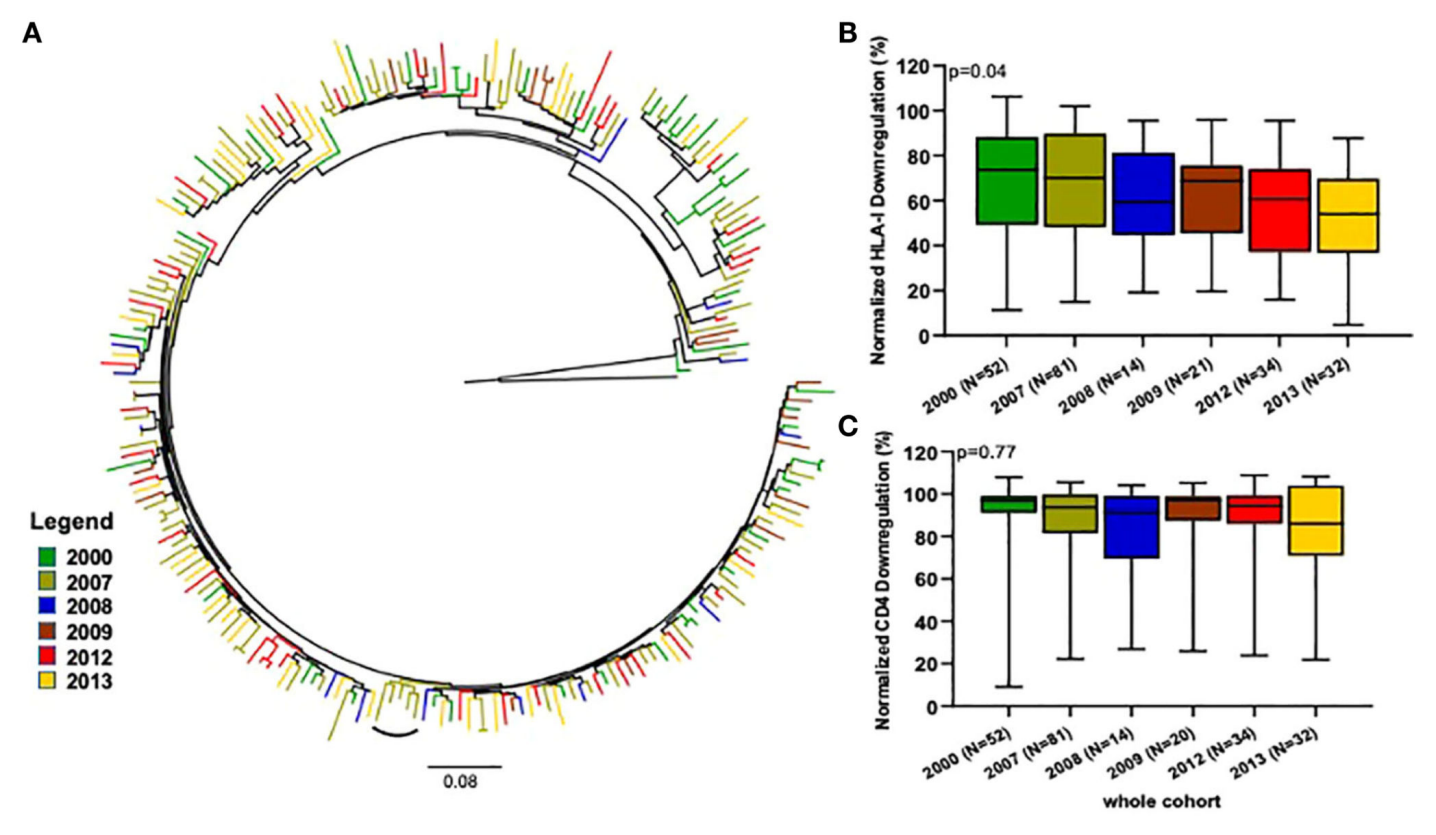
Comparison of Nef-mediated HLA-I and CD4 downregulation over time. **(A)** Represents a maximum-likelihood phylogenetic tree of CRF02_AG *nef* clone sequences according to the year of sampling. The tree was constructed with 1000 full maximum likelihood bootstrap replicates using IQ Tree and using the best fit GTR+I+G nucleotide substitution model (http://iqtree.cibiv.univie.ac.at). The tree was rooted with a sequence belonging to the Diverg./Recomb. lineage. The arc shapes represent a cluster. Sequences from a specific year are represented by a unique color. The arc shapes represent a cluster. The small square identifies identical sequences from two individuals from the same community who share the same viral strain. HLA-I **(B)** and CD4 **(C)** downregulation activities of Nef clones derived from the samples collected in 2000, 2007, 2008, 2009, 2012 and 2013. Horizontal line and error bars represent the median and interquartile range, respectively. We used the Kruskal-Wallis tests to compare Nef functions across sampling years.

It has been previously hypothesized that the evolutionary trajectory of the HIV-1 population is shifting towards attenuation (44). We therefore examined temporal trends in Nef function over time. Overall, there were 52 viruses sampled in 2000, 81 in 2007, 14 in 2008, 21 in 2009, 34 in 2012 and 32 in 2013. We therefore examined temporal trends in Nef function over time. A maximum-likehood (ML) tree revealed no strong evidence of temporal clustering (Figure 3A).

Our Nef functions analyses revealed an overall steady decline in HLA-I downregulation activities from 2000 (median 74% and IQR 49% - 88%) to 2013 (median 54% and IQR 37% - 70%), that reached statistical significance (p=0.04; Kruskal-Wallis test, Figure 3B). In contrast, the CD4 downregulation activity of the viruses remained similar across the studied years (Figure 3C). Of note, the viral loads of these samples displayed a possibly decreasing trend over time (Supplementary Figure 6).

### Nef function between the cosmopolitan cities and remote settings in Cameroon

**Figure 4:**
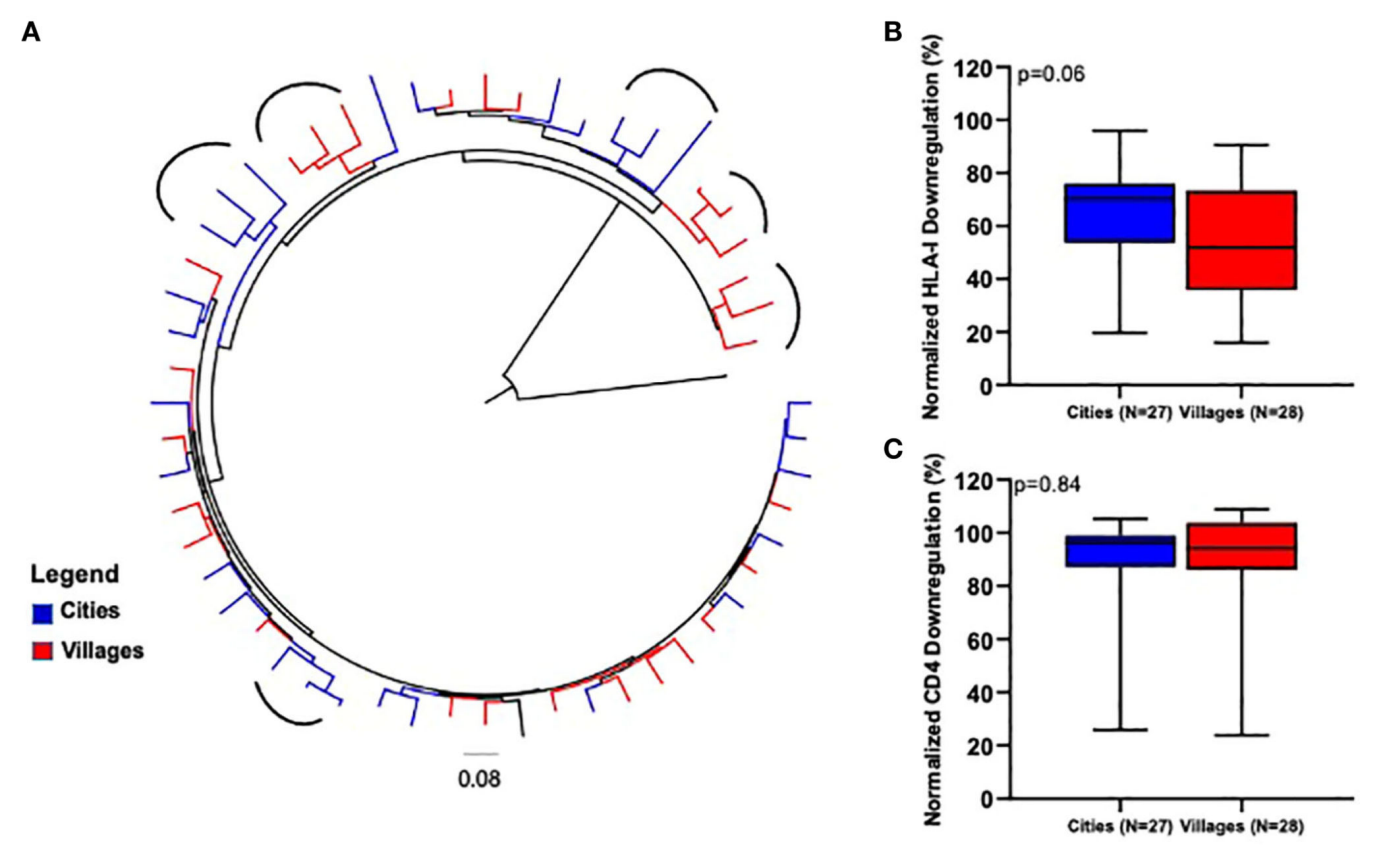
Comparison of Nef-mediated HLA-I and CD4 downregulation between cosmopolitan cities and remote villages. **(A)** represents a maximum-likelihood phylogenetic tree of CRF02_AG *nef* clone sequences according to location sampling. The tree was constructed with 1000 full maximum likelihood bootstrap replicates using IQ Tree and using the best fit GTR+I+G nucleotide substitution model (http://iqtree.cibiv.univie.ac.at). The tree was rooted with a sequence from HIV-1 group P. The arc shapes represent a cluster. Sequences from a specific location are represented by a unique color. HLA-I **(B)** and CD4 **(C)** downregulation activities of Nef clones derived from participants from cosmopolitan cities (2009/2012) versus remote villages (2012). Horizontal line and error bars represent the median and interquartile range, respectively. We used the Kruskal-Wallis tests to compare Nef functions across lineages.

We next compared the HLA-I and CD4 downregulation ability of Nef clones derived from viruses circulating in the cities with those derived from viruses circulating in remote settings. The rationale supporting this is that the two cohorts might have followed different evolutionary trajectories, possibly driven by sexual partner networks likely to be much broader in the highly connected cosmopolitan cities of Yaoundé and Douala than in the much less connected remote villages. For this analysis, we considered samples collected over approximately the same period: 2009 and 2012 (n=27) for the cities and 2012 (n=28) for the remote locations. We began by inferring a maximum-likelihood phylogeny from the 55 clone sequences involved in this comparison, which revealed some evidence of geographical compartmentalization (see round brackets in Figure 4A). Of note, the Nef function analyses revealed a trend towards a higher HLA-I downregulation activity of viruses sampled in the cosmopolitan cities compared to viruses originating from the remote villages, though this was not statistically significant (Mann-Whitney U test, p=0.06, Figure 4B). The CD4 downregulation ability of Nef clones derived from the two environments was similar (Mann-Whitney U test, p=0.78, Figure 4C).

### Sequence determinants of Nef-mediated HLA-I and CD4 downregulation activity

**Figure 5:**
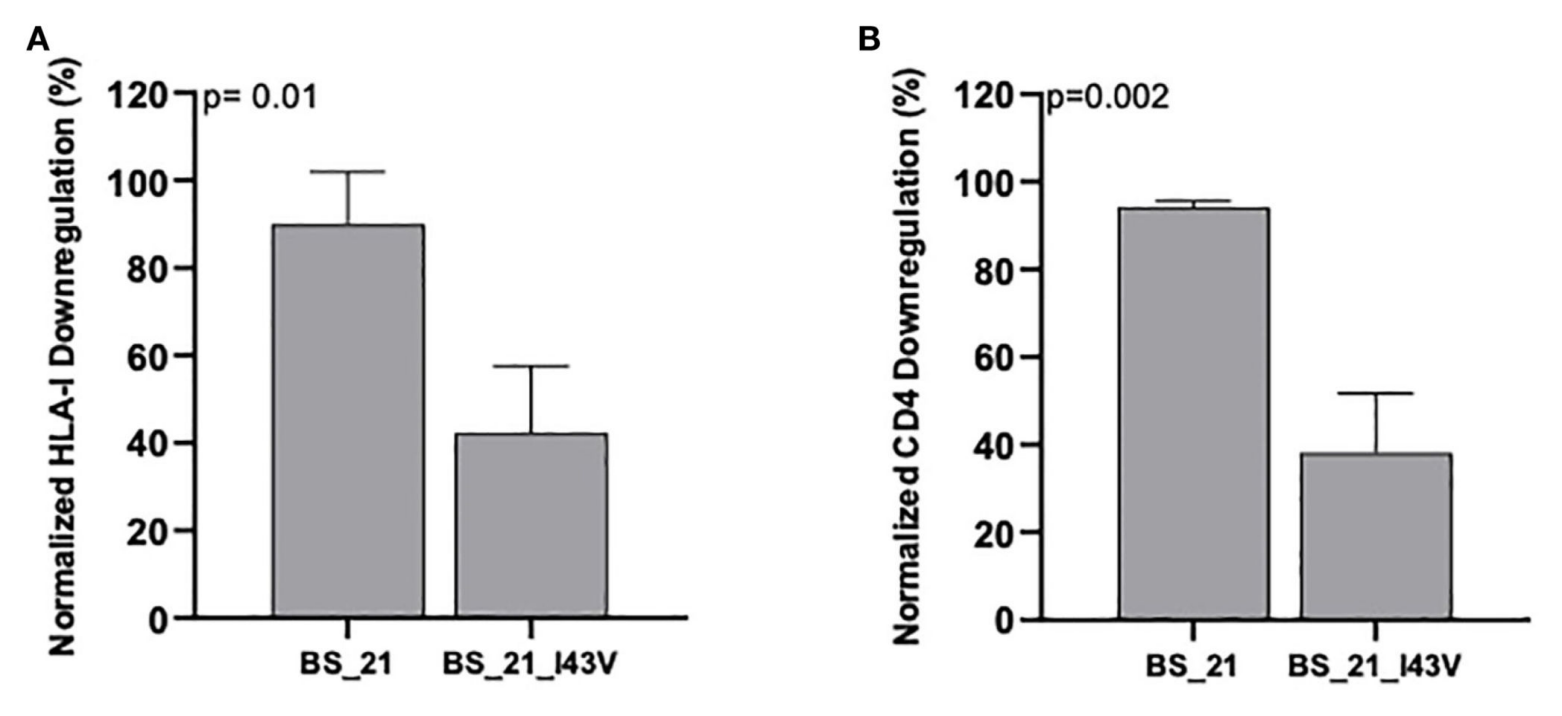
CD4 and HLA-I downregulation activities for HIV Nef mutants. HLA-I downregulation **(A)** and CD4 downregulation **(B)** abilities for the parental sequence (BS21) and its I43V mutant. Bars represent the mean and standard deviation. P-values were calculated using the Unpaired student’s t-test.

We next explored the sequence determinants of Nef HLA-I and CD4 function within CRF02_AG, the dominant circulating variant in Cameroon, by undertaking a codon-by-codon sequence-function analysis. Here, we compared the receptor downregulation function of CRF02_AG sequences harboring (versus not harboring) each amino acid observed at each Nef codon in the dataset (we required a given amino acid to be observed at least five times at that codon to be included in the analysis). In total, 18 amino acids at 15 different Nef codons were associated with differential HLA-I downregulation activity at the exploratory threshold of p<0.05 (Table 2), whereas 13 amino acids at 11 Nef codons were associated with differential CD4 downregulation activity at this threshold (Table 3). Only one of these associations, between Nef codon 43 and HLA-I downregulation, met the q<0.2 threshold for multiple comparisons correction (Table 2). Of note, the Nef I43V substitution was also associated with decreased CD4 downregulation with p<0.03 (Table 3). We therefore selected this mutation for confirmatory testing. To do this, we engineered the I43V substitution into a participant-derived sequence of high similarity to the consensus CRF02_AG sequence by site-directed mutagenesis (participant BS21). As expected, the I43V mutant displayed significantly poorer Nef-mediated HLA-I and CD4 downregulation activity compared to the parental BS21 sequence (p=0.01 and p=0.002, respectively) (Figures 5A, B), consistent with the sequence-function analysis.

## Discussion

The predominance of CRF02_AG (33, 35) despite the tremendous genetic diversity of other HIV-1 group M (HIV-1M) lineages circulating in Cameroon, suggests that most of these other lineages may have failed to reach some required fitness threshold necessarily for wider spread. Hypothesizing that viral lineage-specific differences in Nef’s ability to downregulate HLA-I and CD4 expression may be partially responsible for the differences in prevalence of these lineages, we isolated and functionally assessed unique plasma RNA-derived Nef clones from 234 ART-naive Cameroonians living with HIV. We found that, despite a large degree of within-lineage variation, the ability of Nef to downregulate HLA-I and CD4 molecules did not significantly differ across lineages. We also observed a trend of a higher HLA-I downregulation activity of viruses circulating in cosmopolitan cities versus remote villages. Intriguingly, we observed an overall decrease in HLA-I downregulation activity from 2000 to 2013. Finally, we bioinformatically identified a number of amino acids associated with altered HLA-I and CD4 downregulation activities in CRF02_AG, including the Nef I43V substitution, which we experimentally confirmed to reduce both of these functions.

Our lack of observation of subtype-specific differences in Nef’s ability to downregulate either HLA-I or CD4 molecules is contrary to previous observations by Mann et al. (15) and Turk et al. (28); though it should be noted that these studies did not investigate the specific HIV lineages investigated here, particularly CRF02_AG. One possible explanation could be that evolutionary fitness is unlikely to be the primary determinant of differences in the prevalence of the different major Cameroonian HIV-1M lineages. In support of this interpretation, CRF02_AG has a frequency of >50% and subtype A/A1 a frequency of 2% in Cameroon (35), whereas the prevalence of these lineages is reversed in the Democratic Republic of Congo, with CRF02_AG accounting for 6% of infections and subtype A/A1/A2 accounting for 23% (42). Regional differences in the frequencies of HIV-1M lineages are likely to be more strongly influenced by factors other than differential viral pathogenesis, in particular the relative duration of circulation of a given lineage in a region, and the relative frequencies of variant imports into a region (*i.e*. founder effects) (45). This was well illustrated by Faria and collaborators, who elucidated that CRF02_AG was introduced to Cameroon early on during the pandemic. Since then, at least two CRF02_AG clusters, unique to Cameroon, are evolving and diffusing at different rates. Faria et al. also revealed that outward viral migration was mostly driven by chance exportation events (46).

The diversity of HIV-1M lineages in Cameroon and the pervasive presence of rare highly divergent lineages is consistent with the co-circulation of multiple HIV-1M lineages in Cameroon well before HIV-1 became a pandemic (47). This suggests that there was likely minimal competition between viruses in the different lineages to find and infect new hosts. Therefore, it is reasonable to think that the present-day frequency differences between lineages in Cameroon reflect a composite of slight differences in both the times when lineages first arrived (or emerged) in Cameroon, and the evolutionary fitness these “founder” viruses. Though we did not detect any significant differences in Nef function between HIV-1 subtypes in the present study, we cannot rule out the possibility that such differences are so subtle, and/or within-subtype variations so large, that sample sizes substantially greater than those studied here would be required to detect them. Indeed, a limitation of this study is the relatively limited number of samples representative of lineages other than CRF02_AG.

Interestingly, our results show that while the CD4 downregulation activity of viruses sampled between 2000 and 2013 was similar, there was a steady decline of the HLA-I downregulation ability with the highest activity observed in 2000 and the lowest in 2013. This data is consistent with a prior study (48, 49) that observed that a key Nef activity, namely alteration of TCR signaling, was much lower in Nef clones from Botswana, where the HIV epidemic is older, compared to Nef clones from South Africa, where the epidemic is much younger (48). Our results are also consistent with a study conducted in Japan that reported declining HIV replication capacity over time (49).

Using exploratory sequence-function analyses, we identified several residues in CRF02_AG Nef that were associated with significantly altered HLA-I and/or CD4 downregulation activity, and therefore could potentially affect these Nef functions. From this analysis we identified and experimentally verified that Nef-I43V has a significant negative impact on both HLA-I and CD4 downregulation functions. Although 43V has previously been associated with reduced SERINC5 downregulation activity in subtype B Nef clones (50), it has not to our knowledge previously been associated with altered HLA-I or CD4 downregulation activity,

although it is known that the ability of HIV-1 Nef to downregulate SERINC5 is strongly associated with its ability to downregulate CD4. Structurally, position 43V is found within a disordered region of Nef (residues 35-65) that overlaps with motifs involved in CD4 downregulation (57WL58) and HLA-I downregulation (62EEEE65) (7). The mutation I43V was previously demonstrated to be a viral “escape mutation” selected in CD8+ T cell epitopes restricted by HLA-C*03 (51). There are several other examples of escape mutations in Nef that are associated with reduced Nef function (16, 52, 53), and these may be relevant for HIV-1 attenuation-based vaccine design. Further work will be needed to confirm the effect of Nef 43V in different sequence backgrounds, and to assess the effect of this mutation on Nef expression as well as the mechanism of the defect.

In conclusion, we found that Nef-mediated HLA-I and CD4 downregulation activities varied substantially within, but not between, the HIV-1M subtypes circulating in Cameroon. This suggests that subtype-specific Nef functional variations between lineages is not a major driver of the HIV-1M lineage diversity in Cameroon. However, Nef expression data was not collected and future work is required to validate and extend our findings further.

## Supplementary Material

Supplementary Material

Supplementary Figure Legends

## Figures and Tables

**Table 1:** Participant characteristics (n=234).

	Cosmopolitan cities	Remote villages	p-value
Participant N (total=234)	125	109	
Biological Sex^a^ Male/Female	41/14	17/31	<0.0001
Median age^b^ in years [IQR]^c^	31 [27-38]	32 [26 - 41]	0.79^d^
Median viral load^e^ in log_10_ copies/ml [IQR]	4.4 [3.8-4.9]	4.5 [4.0-5.0]	0.66

a 103 participants with available data

b 101 participants with available data

c IQR = inter-quartile range

d p-values calculated using Mann–Whitney test

e 187 participants with available data.

**Table 2:** Amino acids associated with Nef-mediated HLA-I downregulation function in CRF02_AG Nef clones.

Codon^a^	AA^b^	Cons^c^	% Function with AA^d^	% Function without AA^d^	N with AA^e^	N without AA^e^	Impact ^f^	p-value	q-value
4	K	K	61.8	87.7	122	7	-25.9	0.0298	0.5914
10	L	I	55.0	68.8	30	95	-13.8	0.0413	0.5926
20	M	I	70.2	59.1	64	64	11.1	0.0277	0.5914
25	G	P	51.5	66.9	9	112	-15.5	0.0228	0.5914
32	A	A	66.4	45.0	122	7	21.4	0.0339	0.5914
43	V	I	29.6	65.8	6	123	-36.1	0.0086	0.5810
43	I	I	66.0	36.3	117	12	30.7	0.0002	0.0603
116	H	H	60.2	83.1	106	23	-22.9	0.0107	0.5810
116	N	H	83.1	60.2	23	106	22.9	0.0107	0.5810
149	E	D	90.5	60.8	7	121	29.7	0.0058	0.5810
150	P	P	61.6	83.5	123	5	-22.0	0.0319	0.5914
151	E	A	54.1	62.4	19	109	-8.3	0.0393	0.5926
173	I	M	42.1	66.9	16	111	-24.9	0.0330	0.5914
191	Y	F	46.3	66.4	11	118	-20.0	0.0384	0.5926
192	T	K	83.1	61.2	9	120	21.9	0.0143	0.5810
192	K	K	59.8	71.9	64	65	-12.1	0.0184	0.5914
194	T	I	55.3	68.8	24	105	-13.5	0.0445	0.6032
198	M	L	81.7	60.8	20	109	20.9	0.0274	0.5914

a Numbered according to HXB2.

b Amino acid (AA) associated with altered HLA-I downregulation activity (p<0.05). Only AA observed in a minimum of five sequences were analyzed.

c The consensus amino acid (Cons) at the designated codon from the reference 2021 consensus CRF02_AG Nef sequence from the Los Alamos HIV sequence database.

d The median normalized HLA-I downregulation activity of the Nef clones with (+) and without (-) the amino acid.

e The number of sequences with (+) and without (-) the amino acid.

f The median Nef activity of the clones with the amino acid minus the median Nef activity of those without amino acid.

**Table 3:** Amino acids associated with Nef-mediated CD4 downregulation function in CRF02_AG Nef clones.

Codon^a^	AA^b^	Cons^c^	%Function with AA^d^	% Function without AA^d^	N with AA^e^	N without AA^e^	Impact^f^	p-value	q-value
35	R	Q	87.3	95.5	13	116	-8.2	0.0496	0.6595
43	V	I	65.3	95.0	6	123	-29.7	0.0292	0.6595
65	E	E	95.8	88.4	119	10	7.4	0.0303	0.6595
104	R	K	83.8	95.5	11	118	-11.7	0.0138	0.6595
126	N	N	95.2	52.3	121	8	42.9	0.0105	0.6595
146	V	V	95.2	84.1	119	10	11.1	0.0174	0.6595
146	E	V	84.1	95.2	10	119	-11.1	0.0174	0.6595
150	P	P	93.5	104.4	123	5	-10.9	0.0371	0.6595
151	V	A	99.6	93.4	12	116	6.2	0.0060	0.6595
174	E	E	93.4	99.1	112	15	-5.7	0.0303	0.6595
174	D	E	99.5	93.5	14	113	6.0	0.0453	0.6595
178	K	R	88.7	95.1	10	118	-6.4	0.0477	0.6595
198	M	L	97.9	92.6	20	109	5.3	0.0323	0.6595

a Numbered according to HXB2.

b Amino acid (AA) associated with altered CD4 downregulation activity (p<0.05). Only AA observed in a minimum of five sequences were analyzed.

c The consensus amino acid (Cons) at a particular codon from the reference 2021 consensus CRF02_AG Nef sequence from the Los Alamos HIV sequence database.

d The median normalized CD4 down-regulation activity of the Nef clones with (+) and without (-) the amino acid.

e The number of sequences with (+) and without (-) the amino acid.

f The median Nef activity of the clones with the amino acid minus the median Nef activity of those without amino acid.

## Data Availability

The datasets presented in this study can be found in online repositories. The names of the repository/repositories and accession number(s) can be found in the article/Supplementary Material.
