# Supplementary figures and images for: No detectable differences in Nef-mediated downregulation of HLA-I and CD4 molecules among HIV-1 group M lineages circulating in Cameroon, where the pandemic originated
Source: Front Virol. Author manuscript; Available in PMC 2024 Jun 14. (PMC7616105; doi:10.3389/fviro.2024.1379217)

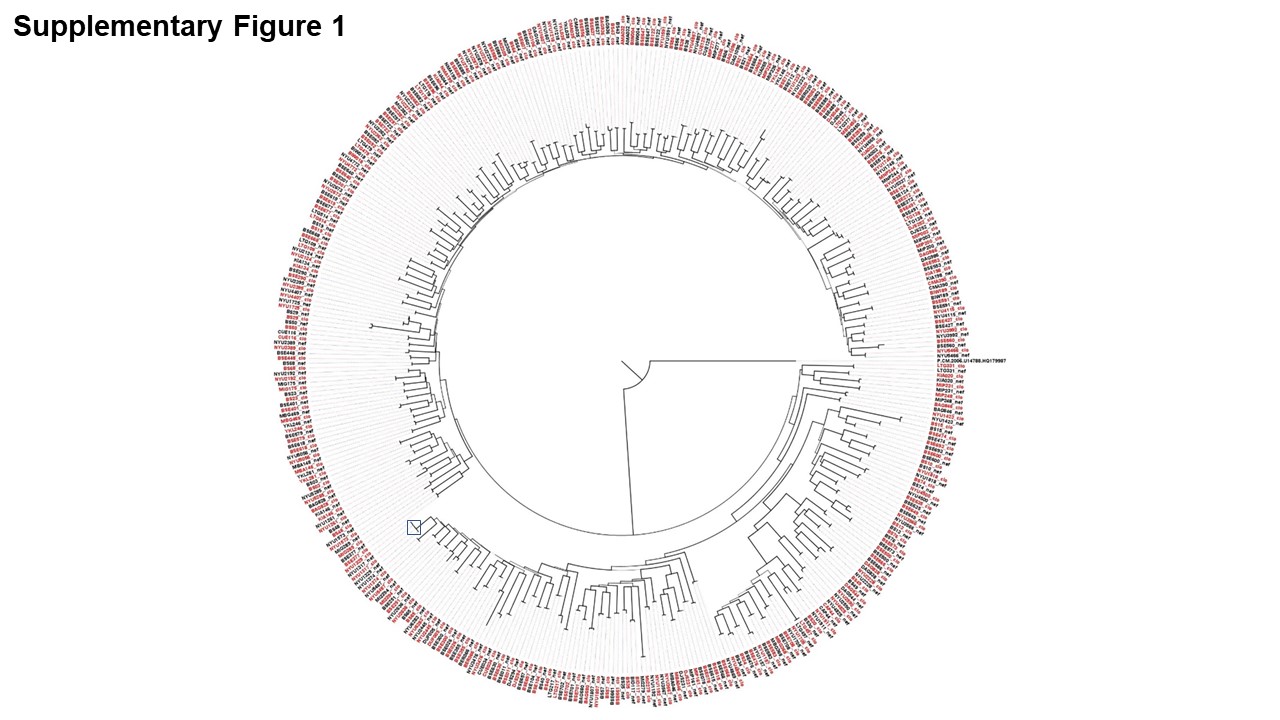

Supplement: Supplementary Material [file EMS196719-supplement-Supplementary_Material.zip › Image 1.jpeg]

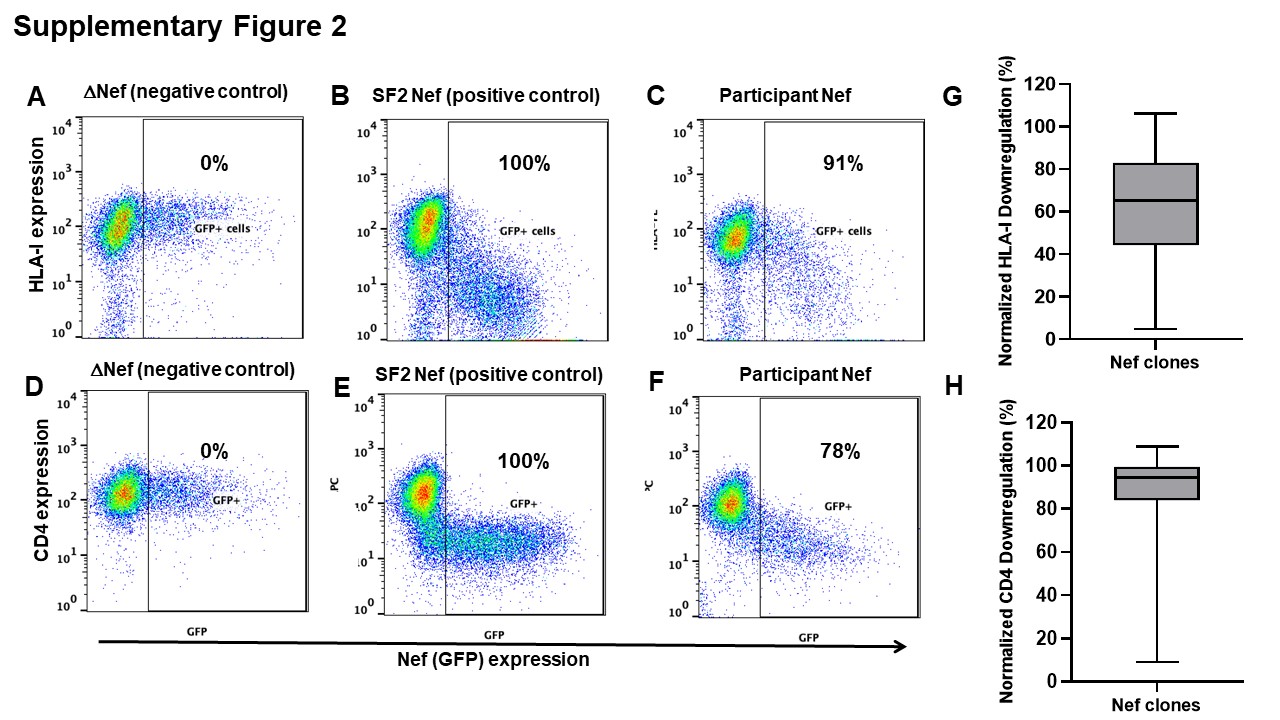

Supplement: Supplementary Material [file EMS196719-supplement-Supplementary_Material.zip › Image 2.JPEG]

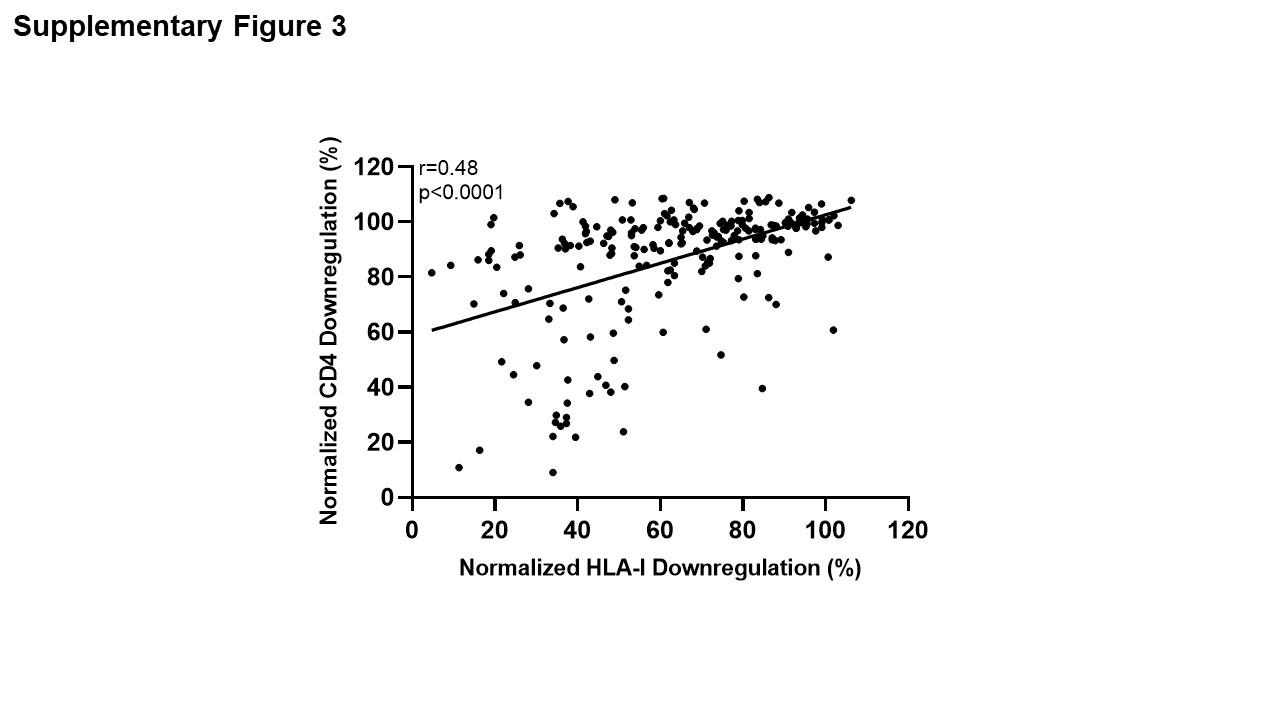

Supplement: Supplementary Material [file EMS196719-supplement-Supplementary_Material.zip › Image 3.JPEG]

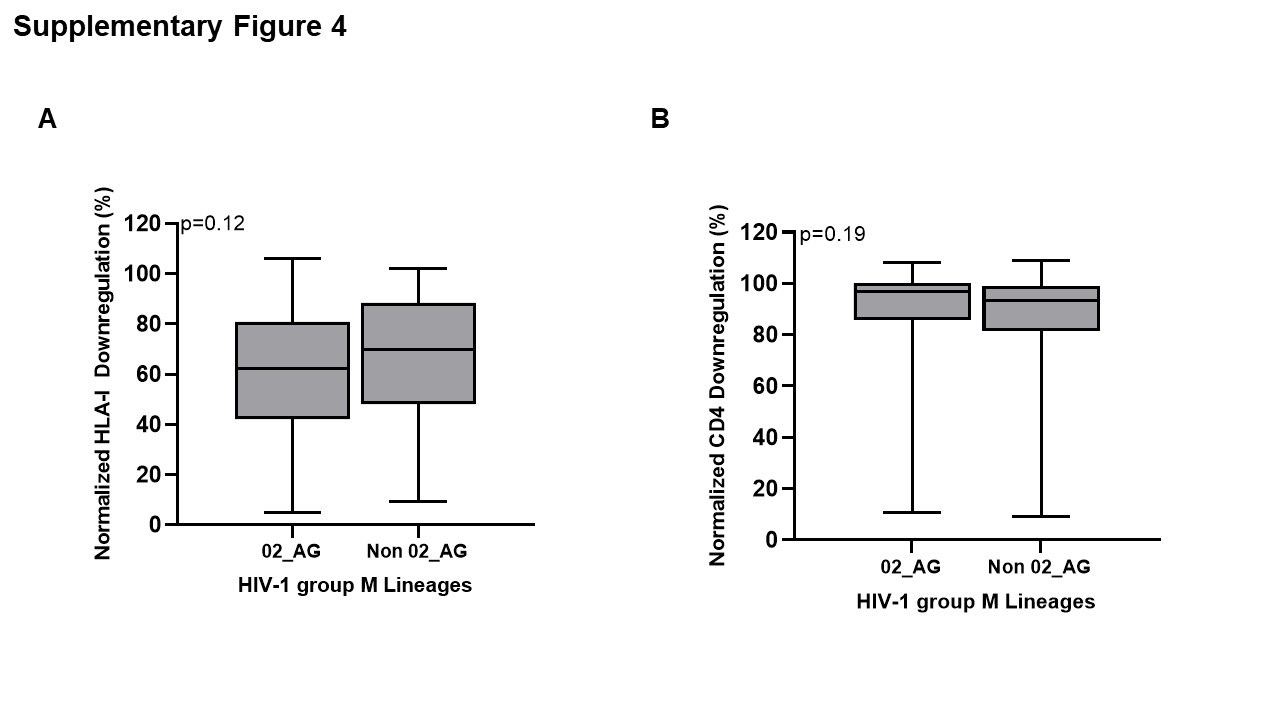

Supplement: Supplementary Material [file EMS196719-supplement-Supplementary_Material.zip › Image 4.JPEG]

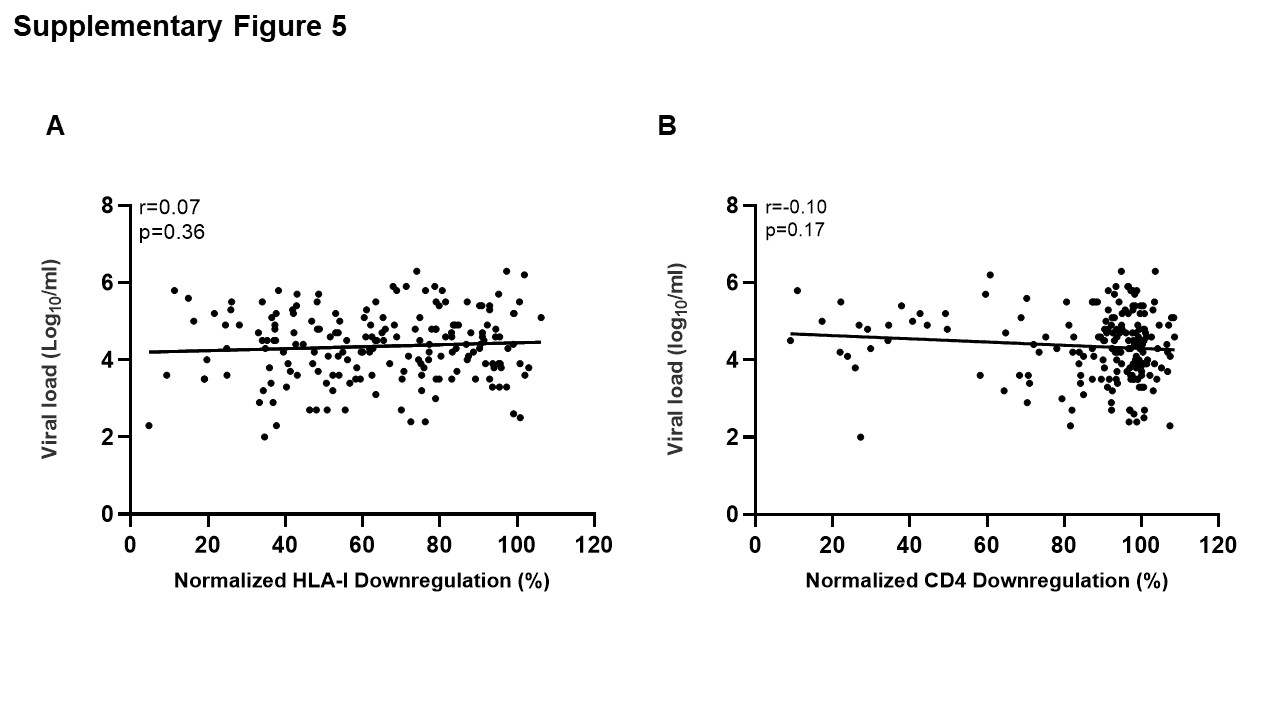

Supplement: Supplementary Material [file EMS196719-supplement-Supplementary_Material.zip › Image 5.JPEG]

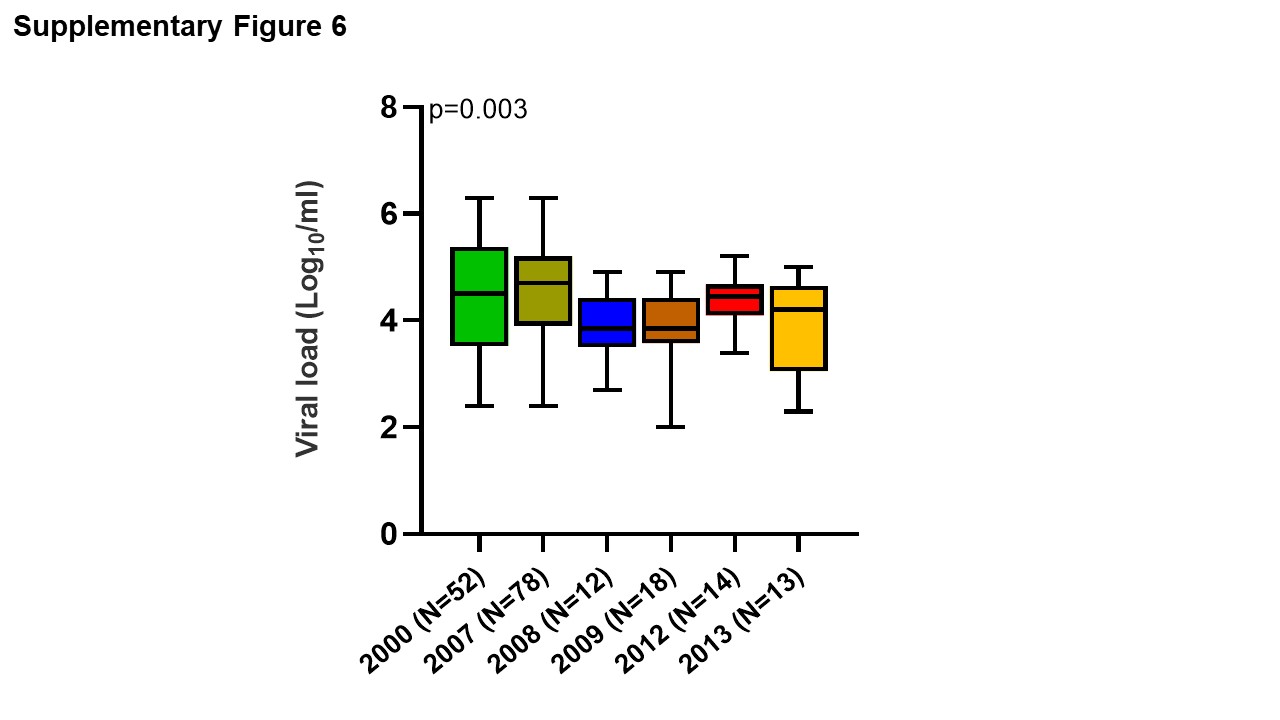

Supplement: Supplementary Material [file EMS196719-supplement-Supplementary_Material.zip › Image 6.JPEG]
