# Supplementary material for: No detectable differences in Nef-mediated downregulation of HLA-I and CD4 molecules among HIV-1 group M lineages circulating in Cameroon, where the pandemic originated
Source: Front Virol. Author manuscript; Available in PMC 2024 Jun 14. (PMC7616105; doi:10.3389/fviro.2024.1379217)
Supplement: Supplementary Figure Legends [file EMS196719-supplement-Supplementary_Figure_Legends.pdf]

s

s

Supplementary material

The Supplementary Material for this article can be found online at: https://www.frontiersin.org/articles/10.3389/fviro.2024.1379217/full#supplementary-material

SUPPLEMENTARY FIGURE 1

Maximum-likelihood phylogenetic tree showing that each clonal *nef* sequence (black label) matches its original bulk sequence (red label). The tree was constructed with 1000 full maximum likelihood bootstrap replicates using IQ Tree and using the best fit GTR+I+G nucleotide substitution model (http://

10.3389/fviro.2024.1379217

iqtree.cibiv.univie.ac.at). The tree was rooted with a sequence from HIV-1 group P. The small square indicates identical sequences from two individuals leaving in the same community who are likely infected with the same virus.

SUPPLEMENTARY FIGURE 2

Nef-mediated cell-surface CD4 **(A–C)** and HLA-I **(D–F)** downregulation. Representative flow cytometry plots of cell-surface expression of CD4 or HLA-I (y-axis) in cells transfected with either an empty plasmid (Δ Nef, negative control), wildtype Nef plasmid (SF2 Nef, positive control) or participant-derived Nef plasmid. The expression of green fluorescent protein (GFP) (x-axis) was used as a marker of Nef-transfected cells. Panels G and H show normalized HLA-I and CD4 downregulation respectively for all lineages combined.

SUPPLEMENTARY FIGURE 3

Correlation between HLA-I and CD4 downregulation activities. Correlation between HLA-I and CD4 downregulation activities for all lineages pooled together. Spearman's r and p-value are shown.

SUPPLEMENTARY FIGURE 4

Comparison of Nef-mediated CD4 and HLA-I downregulation activities in CRF02_AG versus other lineages. HLA-I **(A)** and CD4 **(B)** downregulation abilities between the predominant CRF02_AG lineage and all other lineages included in our study. Bars represent the median and whiskers represent the inter-quartile range for each group. P-values were calculated using the Mann-Whitney U test.

SUPPLEMENTARY FIGURE 5

Correlations between Nef functions and Viral load. Correlation plots for Viral load with HLA-I **(A)** and CD4 **(B)** downregulation activities. Spearman's r and p-values are shown.

SUPPLEMENTARY FIGURE 6

Comparison of Viral Load over time. Viral loads of samples collected in 2000, 2007, 2008, 2009, 2012 and 2013. Horizontal line and error bars represent the median and interquartile range, respectively. Kruskal-Wallis p-value is shown.
